# HIF-1α promotes kidney organoid vascularization and applications in disease modeling

**DOI:** 10.1186/s13287-023-03528-9

**Published:** 2023-11-19

**Authors:** Kexin Peng, Wanqin Xie, Tingting Wang, Yamei Li, Jean de Dieu Habimana, Obed Boadi Amissah, Jufang Huang, Yong Chen, Bin Ni, Zhiyuan Li

**Affiliations:** 1 NHC Key Laboratory of Birth Defect for Research and Prevention, Hunan Provincial Maternal and Child Health Care Hospital, Changsha, Hunan China; 2 Department of Epidemiology and Health Statistics, Xiangya School of Public Health, Central South University, Changsha, Hunan China; 3 CAS Key Laboratory of Regenerative Biology, Guangdong Provincial Key Laboratory of Stem Cell and Regenerative Medicine, Guangzhou Institutes of Biomedicine and Health, Chinese Academy of Sciences, Guangzhou, China; 4 University of Chinese Academy of Sciences, 19 Yuquan Road, Shijingshan District, Beijing, 100049 China; 5 Department of Anatomy and Neurobiology, Xiangya School of Medicine, Central South University, Changsha, China; 6 GZMU-GIBH Joint School of Life Sciences, Guangzhou Medical University, Guangzhou, China; 7 GIBH-CUHK Joint Research Laboratory On Stem Cell and Regenerative Medicine, GIBH-HKU Guangdong-Hong Kong Stem Cell and Regenerative Medicine Research Centre, Guangzhou, China; 8 NHC Key Laboratory of Birth Defect for Research and Prevention, Hunan Provincial Maternal and Child Health Care Hospital, Changsha, China

**Keywords:** HIF-1α, Kidney organoid, Vascularization, Cisplatin

## Abstract

**Background:**

Kidney organoids derived from human pluripotent stem cells (HiPSCs) hold huge applications for drug screening, disease modeling, and cell transplanting therapy. However, these applications are limited since kidney organoid cannot maintain complete morphology and function like human kidney. Kidney organoids are not well differentiated since the core of the organoid lacked oxygen, nutrition, and vasculature, which creates essential niches. Hypoxia-inducible factor-1 α (HIF-1α) serves as a critical regulator in vascularization and cell survival under hypoxia environment. Less is known about the role of HIF-1α in kidney organoids in this regard. This study tried to investigate the effect of HIF-1α in kidney organoid vascularization and related disease modeling.

**Methods:**

For the vascularization study, kidney organoids were generated from human induced pluripotent stem cells. We overexpressed HIF-1α via plasmid transfection or treated DMOG (Dimethyloxallyl Glycine, an agent for HIF-1α stabilization and accumulation) in kidney progenitor cells to detect the endothelium. For the disease modeling study, we treated kidney organoid with cisplatin under hypoxia environment, with additional HIF-1α transfection.

**Result:**

HIF-1α overexpression elicited kidney organoid vascularization. The endothelial cells and angiotool analysis parameters were increased in HIF-1α plasmid-transfected and DMOG-treated organoids. These angiogenesis processes were partially blocked by VEGFR inhibitors, semaxanib or axitinib. Cisplatin-induced kidney injury (Cleaved caspase 3) was protected by HIF-1α through the upregulation of CD31 and SOD2.

**Conclusion:**

We demonstrated that HIF-1α elicited the process of kidney organoid vascularization and protected against cisplatin-induced kidney organoid injury in hypoxia environment.

**Supplementary Information:**

The online version contains supplementary material available at 10.1186/s13287-023-03528-9.

## Introduction

To maintain body-fluid homeostasis, kidneys play a critical role in excreting waste and extra fluid, water-sodium regulation, and acid–base balance. The nephron is the unit of the kidney and is composed of glomeruli featured as podocytes with enriched capillaries, proximal tubule, loop of Henle, and distal nephron. Recently, several innovative protocols have been published for generating kidney organoids from HiPSCs that morphologically resemble kidneys. In 2015, Takasato et al., established a relatively mature and stable kidney organoid differentiation schematic protocol. HiPSCs were successfully differentiated in APEL medium with CHIR99021, FGF9, and Heparin. At the later stage, cells were self-assembled into organoids containing multiple compartments like glomeruli, proximal tubules, and distal tubules within 25 days [1, 2]. Morizane et al., cultured HiPSCs in basal medium (Advanced RPMI 1640 with glutamax) with stepwise modulation of WNT, FGF, and TGFβ signaling to induce kidney organoids within 21 days [3, 4]. Although these novel findings elicited applications in kidney organoids, there are still limitations in current organoids. In 2018, a study compared the kidney organoids generated by the above two protocols at the single-cell transcriptomic level and found that both protocols generated immature kidney organoids with partial gene expression, unlike that of adult kidneys [5].

With the increase of size and culturing time, the core of the organoid cells maintained a hypoxia environment associated with the lack of nutrients in such a harsh condition, making it difficult to maintain their nephron structures, eventually leading to apoptosis [6]. Scientists have tried to overcome these problems by introducing endothelium in kidney organoids. Previous studies demonstrated that kidney organoids derived vasculature via flow [7], modulation of protocols [8], extracellular matrix [9], or transplantation [10]. Despite all these paradigms and interventions, there still are multiple unmet needs. Considering the hypoxia environment in the core of the kidney organoid, we demonstrate a novel paradigm, which is quite essential for kidney organoid vascularization and survival.

HIF-1α is essential for both pathological and physiological processes [11]. HIF-1α is activated and/or overexpressed in the hypoxia environment for angiogenesis via VEGF and VEGFR to promote survival [12]. Taking into consideration the role of HIF-1α in increasing cell survival via vascularization, our study speculates that HIF-1α reveals an essential role in angiogenesis during kidney organoid development. In this study, we introduced HIF-1α into kidney progenitor cells to explore whether it can promote the formation of vascular networks in the kidney organoid.

Kidney organoid is a suitable platform for kidney-related disease modeling. Besides the effect of the vasculature, HIF-1α has been proven to have some beneficial effects on kidney injuries, such as cisplatin-induced renal damage in cultured cell lines and animals [13, 14]. In this study, we firstly detected the effect of HIF-1α on cisplatin-induced kidney organoid injury.

## Methods

### Culture of HiPSCs

All procedures performed in this study involving HiPSCs were approved by the Hunan Provincial Maternal and Child Health Care Hospital Medical Ethics Committee. The study was approved under the project, entitled “Generation of vascularized kidney organoid for disease modeling from human induced pluripotent stem cells” (No. 2021-S074). HiPSCs were generated by reprogramming the urinary cells from a healthy 20–30-year-old individual as described in our previous work [15]. We thawed and cultured human-induced pluripotent stem cells in mTeSR with 10 μM Y27632 on a Geltrex-coated plate for 24 h. Y27632 was removed and mTeSR was changed daily. We passaged HiPSCs every 5–7 days in clumps, using Gentle Cell Dissociation Reagent (Gibco) at split ratios of 1:4 to 1:8.

### Generation of kidney organoids

We cultured HiPSCs on geltrex-coated 6-well plates. After digestion with Accutase, we added mTeSR medium to cells with 10 μM Y27632 for 24 h. Then we replaced the culture medium with 5–8 µM CHIR99021 in Advanced RPMI 1640 supplemented with Glutamax and the cells were further cultured for 72–96 h; then after, the medium was changed to Activin A differentiation medium (10 ng/ml Activin A, 200 ng/ml FGF9, and 1 µg/ml heparin) and cultured for 24 h for the the cells to reach metanephric interstitial stage. The cells were then digested with Accutase, transferred into a fresh medium (3 µM CHIR99021, 200 ng/ml FGF9, and 1 µg/ml heparin), cultured for 1 h, and then the cells were divided into 2D and 3D cultures. 2D cultured cells were transferred into a geltrex-coated plate whereas 3D cultured cells were transferred into a low-attachment 96-well U-bottom plate. all the cells were cultured in a differentiation medium (200 ng/ml FGF9 with 1 µg/ml heparin) for 5 days. The cells self-assembled into organoids (tubules and glomerular structures) after FGF9 and heparin were removed, then we continued to culture these organoids in the basal differentiation medium until the differentiation process was completed. Details of all the reagents used has been provided in Additional file 3: Table S1.

### The overexpression of HIF-1α in kidney progenitor cells

The HIF-1α overexpressing plasmid and mCherry- HIF-1α overexpressing plasmid were designed by Vector Builder Company. HiPSCs were differentiated into kidney progenitor aggregates. On day 10, we performed transfection with mCherry plasmid, mCherry-HIF-1α overexpressing plasmid or HIF-1α overexpressing plasmid using Lipofectamine 3000 reagent. 72 h later, we observed the mCherry signals under confocal microscopy, the transfection efficiency was calculated by the percentage of mCherry positive cells to total cells (DAPI) using Image J software. For DMOG treated group, kidney progenitor cells were treated with 10 μM DMOG for 6 consecutive days. All the organoids were subjected to immunofluorescence analysis to detect the expression of the HIF-1α, CD31, LTL, WT-1, Nephrin, and α-SMA.

### Cisplatin treatment to kidney organoid within hypoxia environment

The HiPSCs were induced into kidney progenitor aggregates and then the HIF-1α overexpression plasmids were transfected into kidney progenitor cells. 4 days later, the organoids were transferred into 1% O_2_ and 5% CO_2_ environment at 37 ℃. 10 μM of cisplatin was used to treat the kidney organoids under hypoxia environment for 48 h, then after, the kidney organoids were harvested, and further analyzed.

### Immunofluorescence analysis of organoids

Kidney organoids featured as podocytes (Nephrin), renal tubules (LTL), and distal tubules (CDH1). Organoids were fixed with 4% paraformaldehyde, permeabilized in Triton-X100 for 10–20 min, and blocked with a blocking buffer (PBS complemented with 3% FBS, 2% BSA, 0.25% Tween 20, and 0.25% Triton-100) for 2 h at room temperature. For the identification of human vascular cells, staining was done using anti-human CD31 (1:100), and anti-smooth muscle actin (1:100) primary antibodies. For the identification of human kidney cells, we adopted human Anti-Nephrin (1:100); human Anti-CDH1 (1:100); human Anti-Wilm's Tumor (1:100), Anti-human HIF-1α (1:100); Anti-human cleaved caspase 3 (1:100); human Anti-SOD2(1:100); Anti-human HO-1 (1:100) primary antibodies (details of the antibodies we used was provided in Additional file 3: Table S1). The organoids were stained with the primary antibodies, incubated overnight at 4 °C on a shaker, and then washed thrice with PBST for 10 min. After that, the organoids were stained with fluorescent secondary antibodies in the dark at room temperature for 2 h and then washed with PBST followed by an optional staining step with renal proximal tubules (LTL) if required. Shortly, the organoids were blocked with LTL blocking solution (Vector labs’ Solution A for 15 min and Solution B for another 15 min), and then incubated with LTL (Vector labs) for 30 min followed by 3 times washing with PBST. Finally, 4',6-diamidino-2-phenylindole (DAPI) dye was added for nuclear staining, and then the organoids were mounted with ProLong™ Gold Antifade Reagent. Fluorescent signal was observed under a Zeiss confocal microscopy.

### QRT-PCR analysis

Total RNA was extracted from human kidney organoids using TRIzol (Thermo Fisher Scientific) according to the manufacturer’s instructions. 1 µg of RNA was used as a template to synthesize cDNA using PrimeScript RT Master Mix Kit (Takara, Tokyo, Japan). Real-time RT–QPCR was performed using TB GREEN PREMIX EX Taq Kit (Takara, Tokyo, Japan). For quantitative real-time RT-PCR (qRT-PCR, 1 µg of RNA was used as a template using the following stepwise amplification setup; 95 °C for 30 s, 40 cycles at 95 °C for 5 s, 60 °C for 34 s, and finally a melt curve from 60 °C to 95 °C. GAPDH housekeeping gene was used as a control. Gene expression was determined by the comparative ΔCT method. The following pairs of gene-specific primers were used (forward and reverse sequence): HIF-1α (F: 5′-TATGAGCCAGAAGAACTTTTAGGC -3′; R:5′-CACCTCTTTTGGCAAGCATCCTG -3′), CD31(F:5′-AAGTGGAGTCCAGCCGCATATC -3′; R:5′-ATGGAGCAGGACAGGTTCAGTC -3′), SOD2 (F:5′-CTGGACAAACCTCAGCCCTAAC -3′; R:5′-AACCTGAGCCTTGGACACCAAC -3′).

### Rendering and analysis of image

We took the bright field photo of the organoids at various differentiation stages using phase contrast microscopy (Olympus) with objectives ranging from 5 to 40X. The confocal analysis was performed by Zeiss LSM 800, LSM 900, and LSM 980 confocal microscopy with objectives ranging from 10 to 40X with spectral lasers of.

405, 488, 561, and 640 nm wavelengths. Confocal Z-stacks were used for either fixed whole or partial kidney organoids. For 3D images, the Z-stacks were employed at the limit of the confocal depth, which was about up to 250 μM for each sample. The 3D reconstruction images were collected using a Zeiss LSM 980 confocal microscope. We collected and analyzed three-dimensional images and rotating movies using Zeiss Zen-Lite software. Vessel percentage area, total junction number, and average vessel length for vascularized kidney organoids were analyzed using Angiotool software [16]. GraphPad Prism (version 9) was used to draw charts and for data analysis. Data were collated and presented as the mean ± SE and analyzed at a 95% confidence level. Statistical significance was represented as *P < 0.05, **P < 0.01, ***P < 0.001 or ****P < *0.0001*.

## Result

### Generation of kidney organoid with glomerular and tubular multicompartment from HiPSCs

**Fig. 1:**
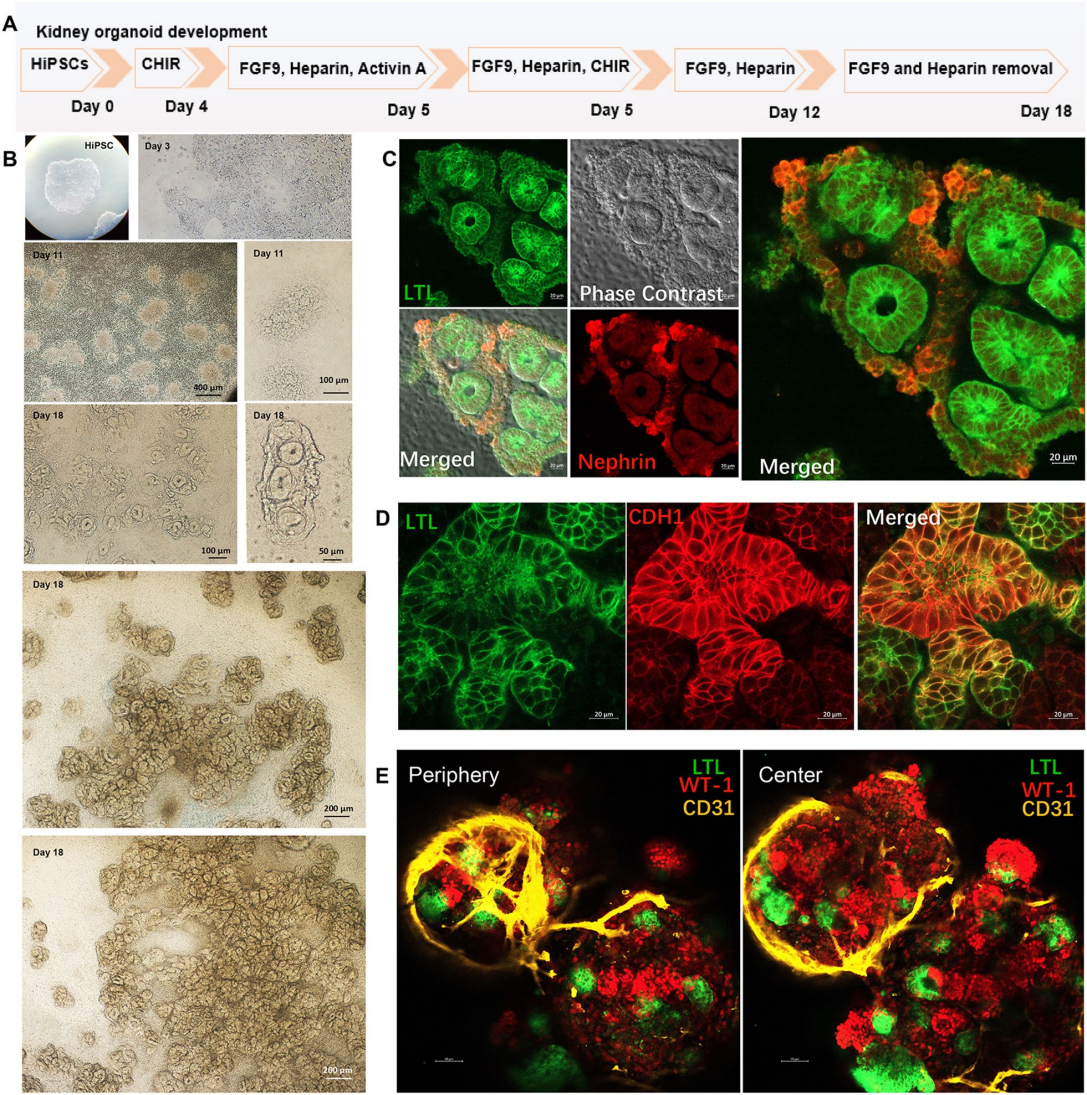
Generation of kidney organoid from human induced pluripotent stem cells. **A** Schematic diagram of kidney organoid differentiation for 18 days, modified from Morizane et al., Takasota et al., and Monteil et al. **B** Phase contrast images from HiPSC to kidney organoid for 18 days. HiPSCs were differentiated to day 7 to kidney progenitor cells. With FGF9 with heparin treatment, kidney organoids matured at day 18 with rough circles. **C** The confocal images for glomerular pars and proximal tubule parts for kidney organoid. The images showed the staining analysis of the proximal tubule (LTL, green), podocyte (nephrin, red), and phase contrast images. **D** The confocal images for proximal tubule (LTL, green) and distal nephrin (CDH1, red). **E** The confocal images for endothelial cells (CD31, yellow), podocytes (Wilms' Tumor, red), and proximal tubule (LTL, green)

Kidney organoid from HiPSCs was generated through an 18-day protocol as previously described as Takasato [1] et al., Morizane et al., [4] and Monteil et al. [17] with slight modifications (Fig. 1A). We cultured the HiPSCs for 4 days to generate primitive streak cells. Then we added the Activin A, FGF9, and Heparin to the culture medium for 1 day to differentiate primitive streak cells into intermediate mesoderm (Fig. 1B). At day 11, the nephron progenitors were positioned in a defined arrangement of initiated glomerular structure and tubular part (Fig. 1B). At day 18, the kidney organoids were generated with defined circles with surrounded compartments (Fig. 1B). We collected organoids. The confocal analysis showed the characteristics of kidney organoid with podocyte (NPHS1 +) and proximal tubule (LTL +) (Fig. 1C). The distal nephron compartments also emerged at day 18. Confocal imaging revealed the formation of kidney-specific tubular structures including proximal tubules (LTL +), and distal nephron (CDH1 +) (Fig. 1D). Endothelium serves an essential role in the renal glomerular filtration barrier. We observed endothelial cells (CD31 +), podocytes (Wilm'sTumor + , WT-1 +), and proximal tubules (LTL +) (Fig. 1E) at the periphery and inside of the kidney organoid, which indicated the typical features of kidney-specific cell types.

### The overexpression of HIF-1α into kidney progenitor aggregates from HiPSCs

**Fig. 2:**
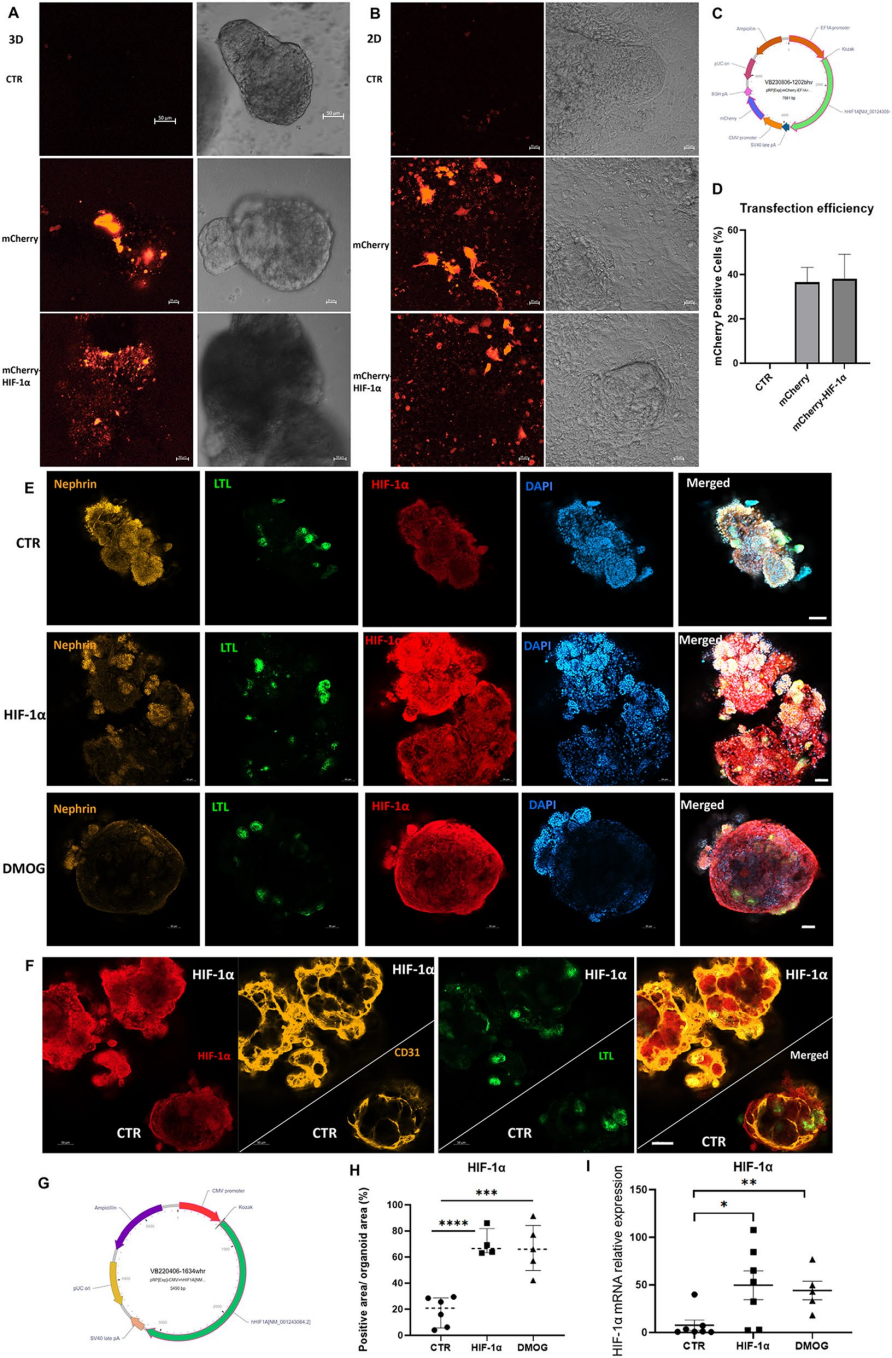
Overexpression of HIF-1α in kidney progenitor aggregates. **A** The mCherry signals in 3D kidney aggregates which were transfected with m-Cherry plasmid or mCherry-HIF-1α overexpressing plasmid for 72 h. **B** The mCherry signals in 2D kidney progenitor cells with the same treatment as above. **C** The design for mCherry-HIF-1α overexpressing plasmid. **D** The transfection efficiency for mCherry positive cells relative to total cells (DAPI) in 2D kidney organoid cells after 72 h transfection. **E** HIF-1α overexpressing organoids or DMOG treated organoids displayed a higher expression of HIF-1α in immune-fluorescence staining (HIF-1α, red), (Nephrin, yellow), (LTL, green) and (DAPI, blue). Scale bar, 50 μm. **F** The fluorescent signals for one HIF-1α overexpressing (HIF-1α, red) kidney organoid (LTL, green) with endothelial cells (CD31, yellow) and one normal kidney organoid. **G** the schematic for HIF-1α overexpression plasmid. **H** the quantification of immunoreactive signals for HIF-1α. Each dot represents the percentage positive area of immunofluorescent signal in each kidney organoid (n = 4–6 per group, **P < 0.01 and ****P < 0.0001 by unpaired t test). **I** HIF-1α relative mRNA expression. (n = 5–7 per group, *P < 0.05 and **P < 0.01 by unpaired t test)

The mCherry-HIF-1α overexpressing plasmid (Fig. 2C) and HIF-1α overexpressing plasmid (Fig. 2G) were designed. We introduced mCherry plasmid, mCherry-HIF-1α overexpressing plasmid or HIF-1α overexpressing plasmid into the 2D and 3D kidney progenitor cells. 72 h later, we observed the mCherry signals at 3D (Fig. 2A) or 2D (Fig. 2B) kidney progenitor aggregates. We detected the transfection efficiency in 2D kidney organoids. There were 36.50% ± 6.7% mCherry positive cells relative to the total cells in mCherry plasmid-transfected organoids (Fig. 2D) and 38.02% ± 11.15% mCherry positive cells in mCherry-HIF-1α plasmid-transfected organoids (Fig. 2D). We also treated kidney progenitor cells with 10 μM DMOG (a cell-permeable prolyl-4-hydroxylase inhibitor, which upregulates HIF-1α) for 6 days. The fluorescent signals (Fig. 2E and H) and mRNA expression (Fig. 2I) of HIF-1α were higher in HIF-1α plasmid-transfected organoids or DMOG-treated organoids than in normal organoids. We captured the immunofluorescent signals for one normal kidney organoid and one HIF-1α plasmid-transfected organoid under the same field of microscopy. Fluorescent signals showed higher HIF-1α signals with more endothelial networks in the HIF-1α plasmid-transfected organoid than in the normal kidney organoid (Fig. 2F).

### HIF-1α promotes the vascularization of kidney organoids via VEGFR

**Fig. 3:**
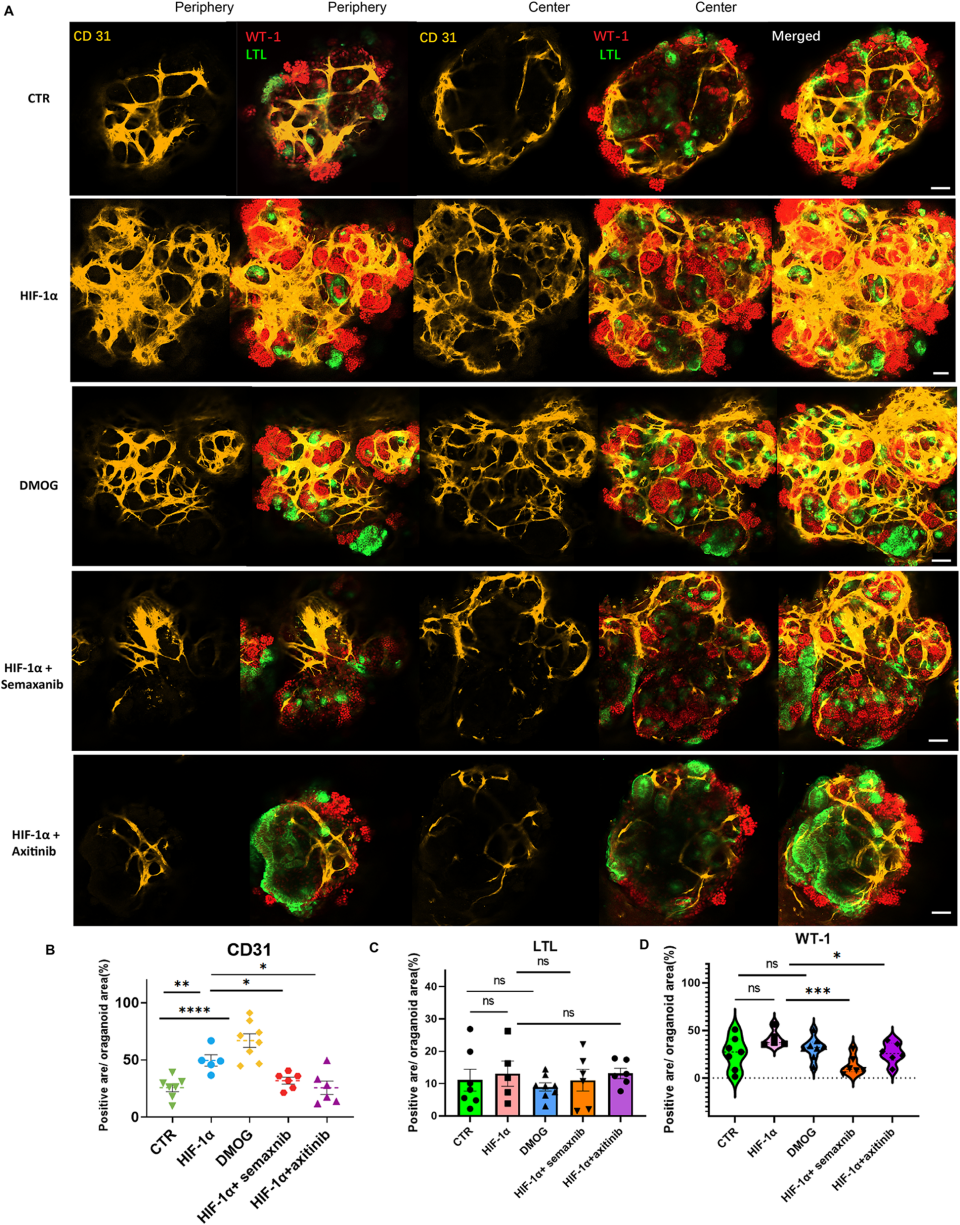
Overexpression of HIF-1α in kidney organoids promotes vascularization. **A** The immunofluorescent result for endothelial networks in kidney organoid. Z-stack confocal analyses were done for kidney organoids (Wilms' Tumor, red; LTL, green) with endothelial networks (CD31, yellow). The data were periphery, center, and merged images of kidney organoid. Five groups of organoids were displayed for control, HIF-1α overexpressing group, DMOG group, HIF-1α with semaxnib group, and HIF-1α with axitinib treated group. Scale bar, 50 μm. **B** The quantification of immunoreactive signals for CD31 (**B**), LTL (**C**), and WT-1 (**D)**. Each dot represents the percentage of immunoreactive signal CD31/LTL/WT-1 positive area to DAPI area in each kidney organoid (n = 5–8 per group, *P < 0.05, **P < 0.01 and ****P < 0.0001 by unpaired t test)

Blood vessels are an essential interface between the circulation systems and kidney environments. During kidney development, metanephric vessels reveal renal blood vessel formation of both renal angiogenesis (the differentiation of capillaries in the embryonic kidney) and vasculogenesis (the in-situ sprouting and growth of endothelial). To detect the effect of HIF-1α on the blood vessel formation in kidney organoid, we performed the Z-stack confocal analysis from the periphery to the core of organoids up to 250 µm, taking as 10–50 µm intervals. All the fluorescent signal layers were merged to display the feature of the overall organoid. The kidney organoid within control groups displayed podocytes (WT-1 +), proximal tubule (LTL +) and several endothelial cells (CD31 +) in the periphery, center, and the overall organoid (Fig. 3A). The confocal analysis revealed that kidney organoids transfected with HIF-1α overexpressing plasmids contained the tubular parts (LTL +) and glomerular compartments (Wilm's Tumor +) which was tightly surrounded with numerous sprouting microvasculature from the periphery to the center of the organoid (Fig. 3A). The merged photo of all layers revealed the overall organoid packed with blood vessel networks in HIF-1α plasmid-transfected organoid (Fig. 3A). Similarly, The DMOG-treated organoid also showed more endothelium in the periphery, center, and the overall kidney organoids than in the normal organoid (Fig. 3A). We analyzed the ratio of CD31 positive area to the DAPI area. The HIF-1α plasmid-transfected organoids or DMOG-treated organoids had an increased percentage of CD31 positive structures than normal kidney organoids (Fig. 3B). To investigate the mechanism underlying HIF-1α-regulated endothelial networks. We treated the HIF-1α plasmid-transfected organoids with semaxanib (a tyrosine kinase inhibitor with a small molecular weight of vascular endothelial growth factor (VEGF) receptor-2) at 10 nM or axitinib (an inhibitor of VEGFR1, VEGFR2, VEGFR3, and PDGFRβ) at 10 nM for 6 days. The semaxanib or axitinib effectively decreased HIF-1α induced endothelial networks at the periphery, center, and overall kidney organoid (Fig. 3A and B). In contrast, there were no detectable differences of the kidney proximal tubule positive structure (the percentage of LTL positive area to organoid area) (Fig. 3C) within these five groups. The podocyte area (the percentage of WT-1 positive area to organoid area) was not changed in HIF-1α plasmid-transfected organoids or DMOG-treated organoids in contrast to normal organoids. However, the semaxanib or axitinib decreased the positive podocyte area compared with HIF-1α plasmid-transfected organoids (Fig. 3D). Considering the tight relationship between podocyte and blood vessel, we speculate that the decreased endothelial cells caused by seaxanib or axitinib could not support podocytes survival, resulting in a reduced podocyte area. To ensure the consistency for the organoid, we analyzed 5 to 8 organoids for each group. And these organoids in each group revealed similar characteristics (Additional file 1: Fig. S1).

**Fig. 4:**
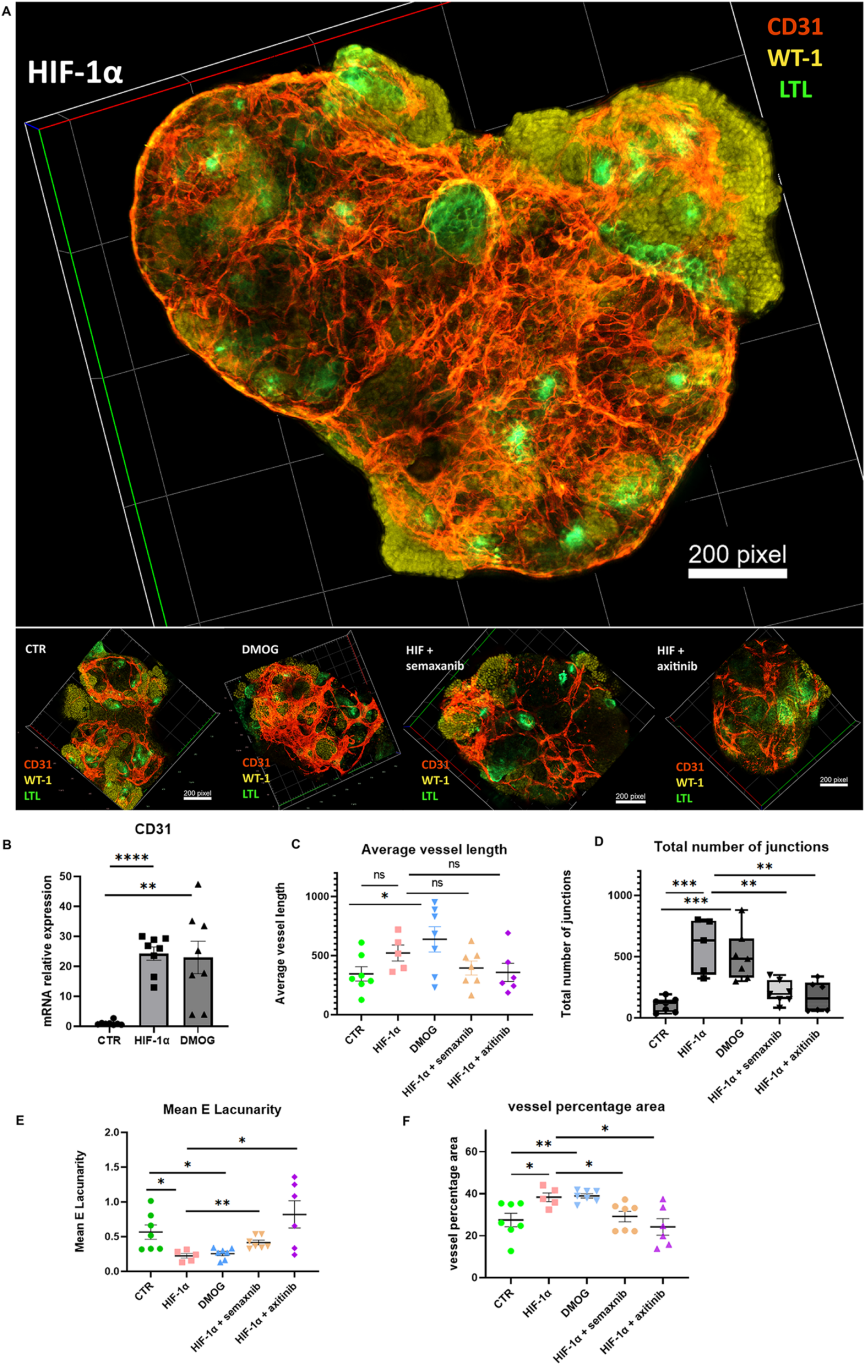
The confocal analysis in HIF-1α-overexpressing kidney organoid. **A** The 3D confocal analysis revealed that transfected HIF-1αkidney organoid positioned glomerular compartments (WT-1, yellow) and tubular parts (LTL, green) packed with endothelial networks (CD31, red). The larger image was HIF-1α transfected group. From left to right in the second line, the four images were controls, DMOG, HIF-1α with semaxanib, and HIF-1α with axitinib treated organoid. **B** The relative mRNA expression of CD31. **C** We use Angiotool analysis software to analyze blood vessels in kidney organoids. The vessel percentage area (**C**), average vessel length (**D**), total number of junctions and Mean E lacunarity (**E**), vessel percentage area (**F**). Each group of samples was displayed. (n = 6–8 per group, *P < 0.05, **P < 0.01, ***P < 0.001 and ****P < 0.0001 by unpaired t test)

To mimic the real organoid architecture, we also performed 3D reconstructions in LSM 980 confocal microscopy (Carl Zeiss). In contrast to the normal organoid, the HIF-1α plasmid-transfected organoid showed the numerous CD31 positive endothelial networks in 3D kidney organoids, and theses vasculature structures sprouted into the entire kidney organoids that morphologically resembled human kidney nephrons enriched of capillaries systems and the vasa microcirculation (Fig. 4A). Video visuals showed the all-layer signals of WT-1 (yellow), CD31 (red) and LTL (green) in the HIF-1α plasmid-transfected organoid (Additional file 2: Fig. S2). The DMOG-treated organoid showed similarly upregulation of endothelium networks (Fig. 4A). The CD31 mRNA expression was significantly increased in HIF-1α plasmid-transfected organoids or DMOG-treated organoids compared with normal organoids (Fig. 4B). The Angiotool analysis was done for these organoids. The DMOG upregulated the average vessel length in kidney organoids (Fig. 4C). The HIF-1α plasmid-transfected organoids or DMOG-treated organoids displayed the elevated vessel percentage area (Fig. 4F), total number of junctions (Fig. 4D), and decreased Mean E lacunarity (Fig. 4E) compared with normal kidney organoids. The 3D construction images showed the semaxanib or axitinib blocked the HIF-1α induced CD31 networks (Fig. 4A), average vessel length (Fig. 4C), total number of junctions (Fig. 4D), vessel percentage area (Fig. 4F), and reversed HIF-1α reduced mean E lacunarity (Fig. 4E). We conclude that the HIF-1α promotes the kidney organoid vascularization through the VEGFR.

### Effect of HIF-1α in kidney organoid on vascular smooth muscle cells

**Fig. 5:**
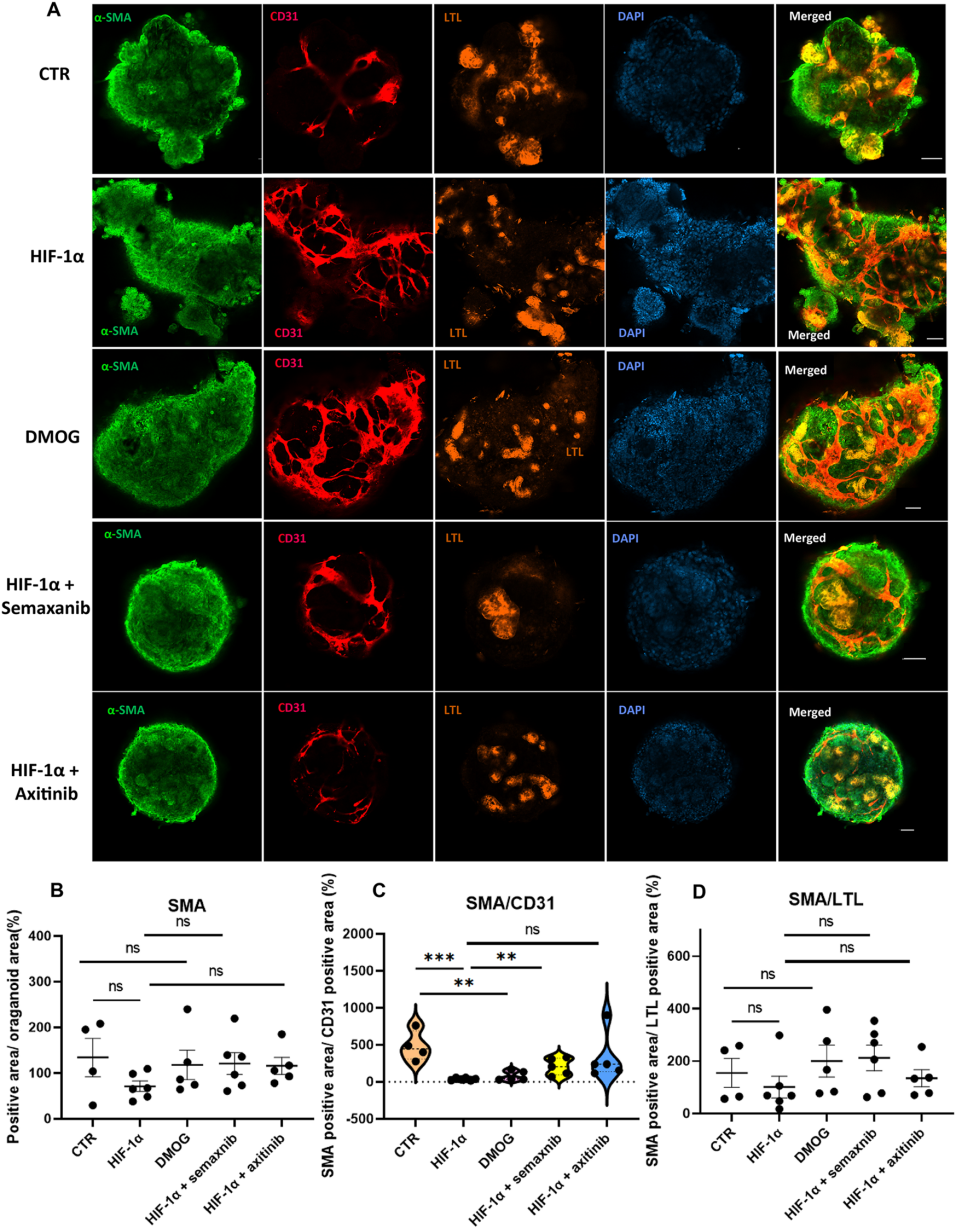
The α-SMA expression in HIF-1α-overexpressing kidney organoid. **A** The immunofluorescent result for vascular smooth muscle cells in kidney organoid. The confocal analysis was done for kidney organoid (LTL, orange) with vascular smooth muscle cells (α-SMA, green) and endothelial networks (CD31, red). The five groups of organoids were the control group, HIF-1α overexpressing plasmid group, DMOG group, HIF-1α with semaxnib group, and HIF-1α with axitinib group. Scale bar, 50 μm. **B** The quantification of immunoreactive signals for α-SMA. Each dot represents the percentage of immunoreactive signal α-SMA positive area to DAPI area in each kidney organoid (n = 4–6 per group, *P < 0.05 by unpaired t test). **C** The ratio of α-SMA positive area to CD31positive area. (n = 4–6 per group, **P < 0.01 and ***P < 0.001 by unpaired t test). **D** The ratio of α-SMA to LTL positive area. (n = 4–6 per group, **P < 0.01 and ***P < 0.001 by unpaired t test)

Vascular smooth muscle cells (VSMC) are essential components in blood vessels. It has also been proven that the vascular smooth muscles serve as a critical regulator in chronic kidney disease [18]. The above results showed the effect of HIF-1α on endothelial cells in kidney organoids. We further investigated the influence of HIF-1α on vascular smooth muscle cells. We analyzed the immunoreactive signals for vascular smooth muscle cell marker (α-smooth muscle actin, α-SMA), CD31, and LTL in kidney organoids. The HIF-1α plasmid-transfected organoids and DMOG-treated organoids revealed a similar α-SMA positive structure with control groups (Fig. 5A and B). The HIF-1α plasmid-transfected kidney organoids exposed to semaxanib or axitinib exhibited a similar α-SMA relative signals (Fig. 5A and B). Since HIF-1α or DMOG increased the endothelium rather than VSMC, we found that the HIF-1α or DMOG decreased the ratio of VSMC-to-endothelium (the positive area of α-SMA relative to the area of CD31) (Fig. 5C). For the ratio of VSMC-to-kidney tubular cells (the positive area of α-SMA relative to the area of LTL), all the five groups showed no differences within these organoids (Fig. 5D).

### HIF-1α protected against cisplatin induced kidney organoid injuries under hypoxia environment

**Fig. 6:**
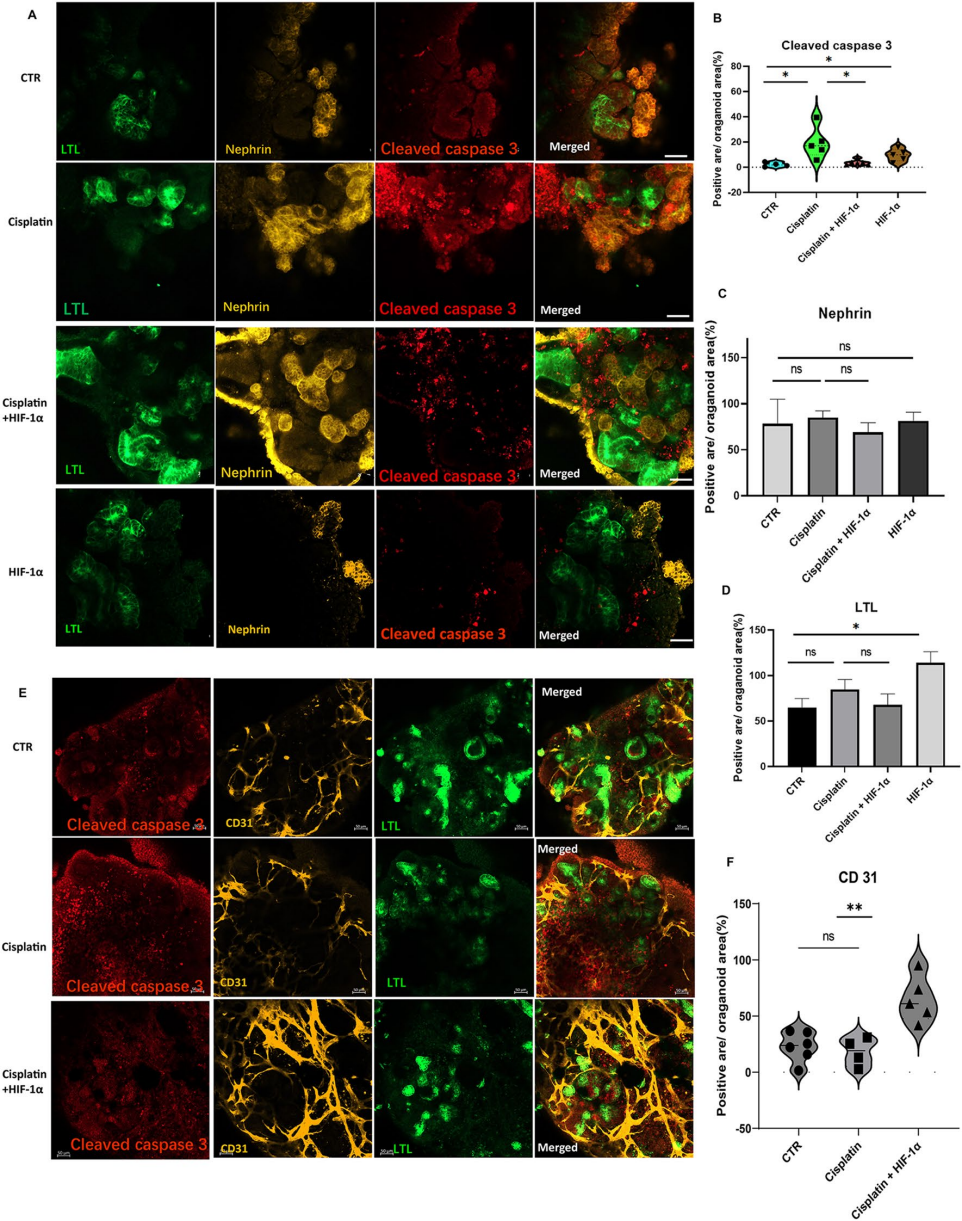
The role of HIF-1α in cisplatin treated kidney organoid. **A** The confocal analysis revealed the immunofluorescent signals of cleaved caspase 3 (red) in kidney organoid (nephrin, yellow, LTL, green). The four groups of organoids were control organoids, cisplatin treated organoids, cisplatin with additional HIF-1α treated organoids, and HIF-1α overexpressing organoids. Scale bar, 50 μm. **E** The confocal analysis revealed the immunofluorescent signals of cleaved caspase 3 (red) and CD31 (yellow) in kidney organoid (LTL, green). Scale bar, 50 μm. **B-D** and **F** The quantification of immunoreactive signals for Cleaved caspase 3 (**B**), Nephrin (**C**), LTL (**D**), CD31 (**F**). Each dot represents the percentage of Cleaved caspase 3 (**B**), Nephrin (**C**), LTL (**D**), CD31 (**F**) positive area to DAPI area (n = 4–5 per group, *P < 0.05 by unpaired t test)

It was proved that HIF-1α had beneficial effects on kidney injury in both cell lines and animals [13, 19]. But the effect of HIF-1α on cisplatin-induced kidney organoid injury has not been investigated. To detect the role of HIF-1α on kidney organoids, we transfected HIF-1α plasmids to kidney progenitor aggregates. Four days later, the kidney organoid was transferred into a hypoxia environment with Cisplatin treatment for 48 h. The hypoxia environment and cisplatin did not destroy the structure of podocyte (nephrin) and tubular parts (LTL) (Fig. 6A). The cisplatin-treated organoids showed no changes for positive nephrin structures (Fig. 6C) and LTL (Fig. 6D) compared with normal organoids. To further confirm the effect of cisplatin-induced cell apoptosis, we detected the cleaved caspase 3 immunofluorescent signals and found that the cisplatin increased the cleaved caspase 3 and this increase was blocked by HIF-1α treatment (Fig. 6A, B). The HIF-1α plasmid-transfected organoids without cisplatin revealed slightly higher cleaved caspase 3 signals compared with control groups (Fig. 5A); we thought that these elevated cleaved caspases 3 signal might be induced by HIF-1α related injury pathways, such as HIF-1α/notch signaling ways. It has been proven that blocking the HIF-1α/Notch 1 signaling impedes kidney injuries [20]. But in our experiments, these injury-related pathways possessed limited effects, the major role of HIF-1α is still protective. To our surprise, the HIF-1α plasmid-transfected organoids had higher LTL immunoreactive signals than the normal kidney organoids, and we speculated that HIF-1α promoted proximal tubule differentiation during the hypoxia environment. We detected the CD31 positive structure in cisplatin treated kidney organoids. We found that the cisplatin did not change the CD31 relative structure. However, the HIF-1α overexpressing organoids exposed with cisplatin had more endothelium than the cisplatin treated kidney organoid (Fig. 6E and F).

### HIF-1α reversed cisplatin reduced SOD2 in kidney organoids under hypoxia environment

**Fig. 7:**
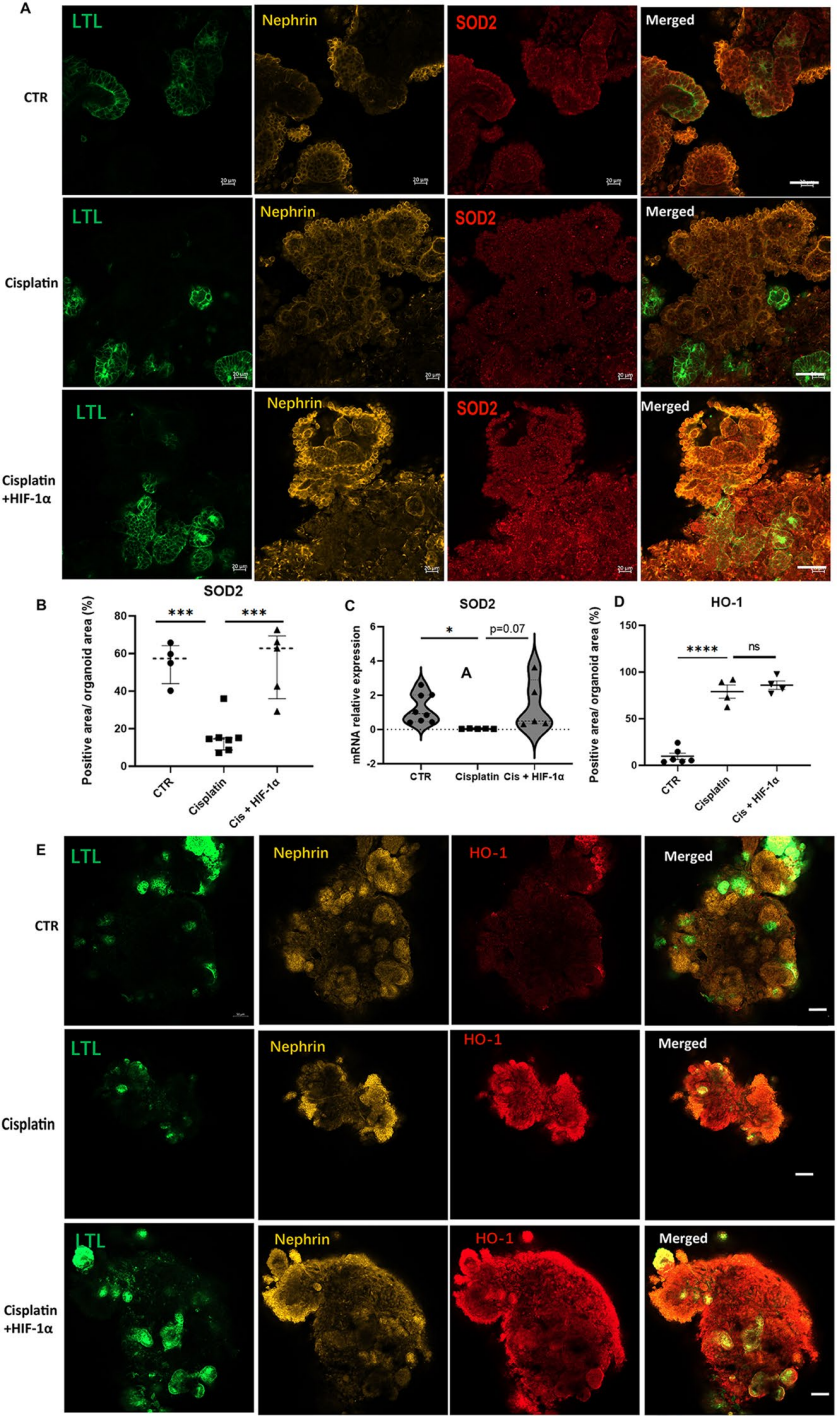
The SOD2 or HO-1 expression in cisplatin treated kindey organoid. **A** The confocal analysis revealed the immunofluorescent signals of SOD2 (red) in kidney organoid (nephrin, yellow; LTL, green). Scale bar, 50 μm. **C.** The relative SOD2 mRNA expression. **B** and **D**. The quantification of immunoreactive signals for SOD2 (**B**)/ HO-1 (**D**). Each dot represents the percentage of immunofluorescent signal SOD2 (**B**)/ HO-1(**D**) positive area/DAPI area in each kidney organoid (n = 4–7 per group, ***P < 0.001 and ****P < 0.0001 by unpaired t test). **E** The immunofluorescent analysis showed the signals of HO-1(red) in kidney organoid (nephrin, yellow, LTL, green). The three groups were CTR, cisplatin and cisplatin with HIF-1α.Scale bar, 50 μm

Mitochondrial superoxide dismutase (SOD2) is an enzyme that protects against oxidative damage in mitochondria which generates energy. It is a key component of the metabolic machinery in the mitochondrial matrix to handle reactive oxygen species (ROS). In this study, we observed that the SOD2 relative positive area (Fig. 7A and B) and relative mRNA expression (Fig. 7C) were decreased by cisplatin and this downregulation was reversed by HIF-1α. Heme oxygenase-1 (HO-1) is expressed in response to stimulations to degrade heme, which generates carbon monoxide((CO), biliverdin, and other biologically active catabolites. In kidney organoids, HO-1 positive area was upregulated by cisplatin, but HIF-1α does not influence HO-1 relative expression (Fig. 7D and E). We conclude that the HIF-1α protected from cisplatin-induced kidney organoid apoptosis via upregulation of SOD2 under hypoxia environment.

## Discussion

Kidney organoids generated from HiPSCs hold substantial potential applications in cell transplanting therapy, drug screening [21, 22] and disease modeling such as COVID-19 [23] or polycystic kidney disease (PKD) [24–26]. But these applications were hindered since the kidney organoid generated in vitro displays immaturity, variability, and limited scale. Long-term cultured 3D kidney organoids were hard to maintain their cellular compartments and characteristics with the existence of internal hypoxia and lack of vasculature. Blood vessel served an essential role in the kidney. The glomerular endothelium, which was fenestrated, involves the sieving characteristics of the glomerular filtration barrier and the podocyte's stability. The fenestrated microvascular cells in peritubular capillaries transport reabsorbed elements and were involved in epithelial cell function. It was of great interest to investigate whether in vitro-generated renal structures have the potential to generate vasculature to improve the maturity and stability of kidney organoids [27]. Researchers had achieved the vascularization of organoids for several years through various methods. (1) Fluid chips promote the vascularization of organoids. In 2019, a study found a correlation between fluid stress and the vascularization of kidney organoids. Kidney organoids were fixed on the matrix of the chip. The differentiation medium flowed into the organoids on the chip and effluence out with multiple cycles, which promoted the generation of the vascular network of kidney organoids [7]. (2) Another study confirmed that adding CHIR99021 to the kidney organoids for 10 days at the later stage of differentiation stimulated podocytes to secrete vascular endothelial growth factor A (VEGFA), which formed the vasculature [8]. (3) Extracellular cellular matrix for kidney organoid vasculature [9]. (4) Rabelink et al., employed intracelomic transplantation into chicken embryos to generate vasculature in kidney organoids [10]. (5) hETV2 created essential links in angiogenesis [28]. These significant vascularization schemes enable the organoids packed with endothelial cells. However, these methods still leave multiple unmet needs. A defined vascularized paradigm requires several features. (1) well-differentiated 3D kidney organoids, especially the core of the organoid should be well vascularized and developed. (2) kidney organoids were tightly surrounded with entire and evenly vascular networks systems, (3) be highly reproducible, stable, and reliable, (4) co-existence of various kidney cell types, (5) be widely used in applications in drug screening, disease modeling, and cell therapy. Here, we introduced the HIF-1α into the differentiating kidney progenitor aggregates to demonstrate a novel vascularization method for kidney organoids, which particularly met these requirements.

Kidney organoids were tight 3D cell spheres with an internal hypoxia environment. In this study, we successfully generated 2D and 3D kidney organoids (Fig. 1 A–E). Then, the HIF-1α overexpressing plasmid was delivered into kidney progenitor cells (FIg. 2A–I); the DMOG treatment was also performed continuously for 6 days (Fig. 2E–I), resulting in the formation of kidney organoids surrounded by blood vessels. These HIF-1α plasmid-transfected or DMOG-treated organoids showed entire blood vessel systems packed into the nephron units, mimicking the organ architecture (Figs. 3 and 4).

Our study demonstrates this novel paradigm to generate kidney organoids with endothelium through HIF-1α, which is highly reproducible and comprises various cell types with podocyte, proximal tubule, distal nephron, endothelium, and VSMC. Therefore, our vascularized kidney organoid cultured system may provide a reliable paradigm to investigate kidney diseases that are related to renal micro vasculature, such as diabetic nephropathy.

Several studies reported that HIF-1α stimulated the formation of endothelial cells through VEGFA receptors. We further explored whether HIF-1α promoted the formation of the vascular network in the kidney organoid via VEGFR1, VEGFR2, and VEGFR3. So, we treated the HIF-1α transfected kidney organoid with semaxanib or axitinib (Semaxanib reversibly blocks ATP binding to the tyrosine kinase domain of VEGFR2; axitinib is a tyrosine kinase inhibitor against VEGFR1, VEGFR2, and VEGFR3.) We observed that the increase of vasculature via HIF-1α in kidney organoids was effectively blocked by semaxanib and axitinib (Figs. 3 and  4). Both semaxanib and axitinib blocked the VEGFR2. We confirmed the role of VEGFR-2 in HIF-1α induced the endothelium. Further study could be performed to confirm the role of specific VEGFR1 or VEGFR3 in the HIF-1α/VEGFR pathway during angiogenesis in kidney organoids. Vascular smooth muscle cells were crucial components in blood vessels and play an essential role in kidneys. We detected a relatively α-SMA positive area in vascularized kidneys. We did not observed differences of α-SMA positive structures in the HIF-1α overexpressing organoids or DMOG-treated group compared with normal kidney organoids. In kidney organoids, HIF-1α induced endothelial networks rather than vascular smooth muscle cells.

HIF-1α is a protective factor in kidney injuries. Numerous studies proved that the HIF-1α protected against early chronic kidney diseases [13], acute kidney injuries [29], cisplatin-induced apoptosis [30], vascular damage, and impaired circulation in animal and cell lines study [14, 31]. The human kidney organoid served as a new platform for drug screening and disease modeling. Gupta. N. et al. [21], and Li, Z. et al. [32], made great efforts to elucidate cisplatin-induced injuries in kidney organoids. Cisplatin is a widely used chemotherapeutic drug that is used to treat cancers but with side effects that initiate kidney injury via oxidative DNA damage and cell cycle arrest, contributing to cell death. The accumulation of cisplatin in the kidney leads to apoptosis of renal cells, inflammatory response, and oxidative stress. This injury plays an essential role in the changes of phenotype exerted on epithelial cells with subsequent loss of kidney function [33]. Caspase-3, a cysteine-aspartic acid protease, is an essential zymogen in cell apoptosis and is activated by cleavage from initiator caspases in the process of apoptotic flux [34]. Less is known about the role of HIF-1α in cisplatin-treated kidney organoids. We firstly observed the protective effect of HIF-1α in cisplatin-induced kidney organoid apoptosis. The upregulation of cleaved caspase 3 was blocked by HIF-1α plasmid transfection. In this study, we treated the kidney organoid under 1% hypoxia environment with 10 μM cisplatin for 48 h. This treatment period was not as long as other studies (Gupta et al. [21],) where kidney organoids were treated with cisplatin with long-term treatment for over 2 weeks, which led to damaged kidney organoid structure. In our short-period cisplatin-treated experiment, the kidney organoid podocyte (Fig. 6A and C), tubular part (Fig. 6A and D) and endothelium (Fig. 6E and F) were not destroyed, but the apoptosis (cleaved caspase 3) was increased and successfully blocked by HIF-1α. Our study confirmed the role of HIF-1α in cisplatin-induced kidney organoids, which provided an injured kidney organoid platform for HIF-1α related drug therapy.

Cisplatin treatment did not decrease or destroy the CD 31 positive structure in our study (Fig. 6E and F). But we found that the HIF-1α overexpressing organoids exposed with cisplatin increased CD31 positive structures compared with cisplatin treated organoids, it indicated that the protection of HIF-1 alpha on cisplatin induced kidney organoid injury was due to the upregulation of endothelium.

Oxidative stress is the primary risk factor in cisplatin-induced kidney injuries. HIF-1α is a critical regulator in oxidative stress [13, 35]. SOD2 protects against oxidative damage to handle ROS. HO-1 is produced to degrade heme and reduce CO. Both SOD2 [36] and HO-1 [37, 38] were regulated via HIF-1α. Our study observed SOD2 and HO-1 positive area in cisplatin-treated kidney organoids. We found that HIF-1α reversed the cisplatin-reduced SOD2. However, the upregulation of HO-1 by cisplatin was not further increased by HIF-1α. In conclusion, we confirmed that the HIF-1α efficiently blocked the cisplatin-induced oxidative stress via SOD2 regulation.

Our study, however, was faced with a few limitations. Several studies transplanted the organoid to immunodeficient mice to verify the organoid functions in vivo [9, 22, 39]. A Study showed that transplanting kidney progenitor cells under a renal capsule effectively prolonged the survival time for the cisplatin-treated immunodeficient mice [32]. In our experiment, we did not perform the in vivo study for function analysis of vascularized kidney organoids. Single-cell RNA sequencing has also been displayed in numerous studies to verify the maturation and cell-specific types in kidney organoids [9]. Our study was limited by the lack of single-cell RNA sequencing and analysis. Several studies observed cellular organelles in the kidney organoid on telecom electron microscopies (TEM)^40^. We did not perform the TEM analysis for vascularized kidney organoids. Despite these limitations, we are confident our results would have reached the same conclusions due to the evidence deduced from several previous studies.

## Conclusion

In conclusion, we developed kidney organoids with entire vascular systems by introducing HIF-1α into kidney aggregates. Our study indicated that the HIF-1α played a critical role in kidney organoid vascularization. Furthermore, we found that VEGFR was required for HIF-1α induced organoid angiogenesis. Our study showed that kidney organoid serves as an advanced platform for disease modeling. The study also proved that HIF-1α was a protective agent against cisplatin-induced kidney organoid apoptosis via the upregulation of CD31 and SOD2. Our study confirmed that HIF-1α revealed an essential role in kidney organoid vascularization and cisplatin-induced kidney organoid injuries.

### Supplementary Information

**Additional file 1**: **Fig. S1**. Overexpression of HIF-1α in kidney organoid promotes vascularization through VEGFR. The immunofluorescent results of endothelial networks for each kidney organoid in Fig 3. The Five groups were controls, HIF-1α, DMOG, HIF-1α with semaxanib, and HIF-1α with axitinib. The images were kidney organoids (Wilms' Tumor, red; LTL, green) with endothelial networks (CD31, yellow).

**Additional file 2**: **Fig. S2**. The movie for 3D vascularized kidney organoid with HIF-1α overexpression. Z-stack confocal analysis were done for vascularized kidney organoid with podocytes (Wilms' Tumor, yellow), endothelial networks (CD31, red) and proximal tubule (LTL, green).

**Additional file 3**: **Table S1**. The details of the companies and catalog number of cell culture materials and antibodies.

## Data Availability

All data generated or analyzed during this study are included in this published article and its supplementary information files. Additional experimental details and more detailed data used or analyzed during the current study are available from the corresponding author upon reasonable request.

## Abbreviations

HIF-1α: Hypoxia-inducible factor-1 α
HiPSCs: Human pluripotent stem cells
DMOG: Dimethyloxallyl Glycine
VEGFR: Vascular endothelial growth factor receptor
HUVEC: Human Umbilical Vein Endothelial Cells
SOD2: Superoxide Dismutase 2
FGF9: Fibroblast Growth Factor 9
TGF-β: Transforming growth factor-β
hETV2: Human ETS variant 2
HO-1: Heme oxygenase 1
2D: Two-dimensional
3D: Three-dimensional
GFP: Green fluorescent protein
PECAM-1: Platelet Endothelial Cell Adhesion Molecule-1
LTL: Lotus Tetragonolobus Lectin
WT-1: Wilms' tumor gene 1
α-SMA: α-Smooth muscle actin
BSA: Bovine serum albumin
FBS: Fetal bovine serum
PBST: Phosphate buffered saline with Tween-20
DAPI: 4',6-Diamidino-2-phenylindole
qRT-PCR: Real-Time Quantitative Reverse Transcription PCR
CHIR: CHIR99021
PDGFRβ: Platelet-derived growth factor receptor beta
ROS: Reactive oxygen species
CO: Carbon monoxide
TEM: Transmission Electron Microscopy
