# Supplementary figures and images for: HIF-1α promotes kidney organoid vascularization and applications in disease modeling
Source: Stem Cell Res Ther. 2023 Nov 19;14:336. doi: 10.1186/s13287-023-03528-9 (PMC10659095; doi:10.1186/s13287-023-03528-9)

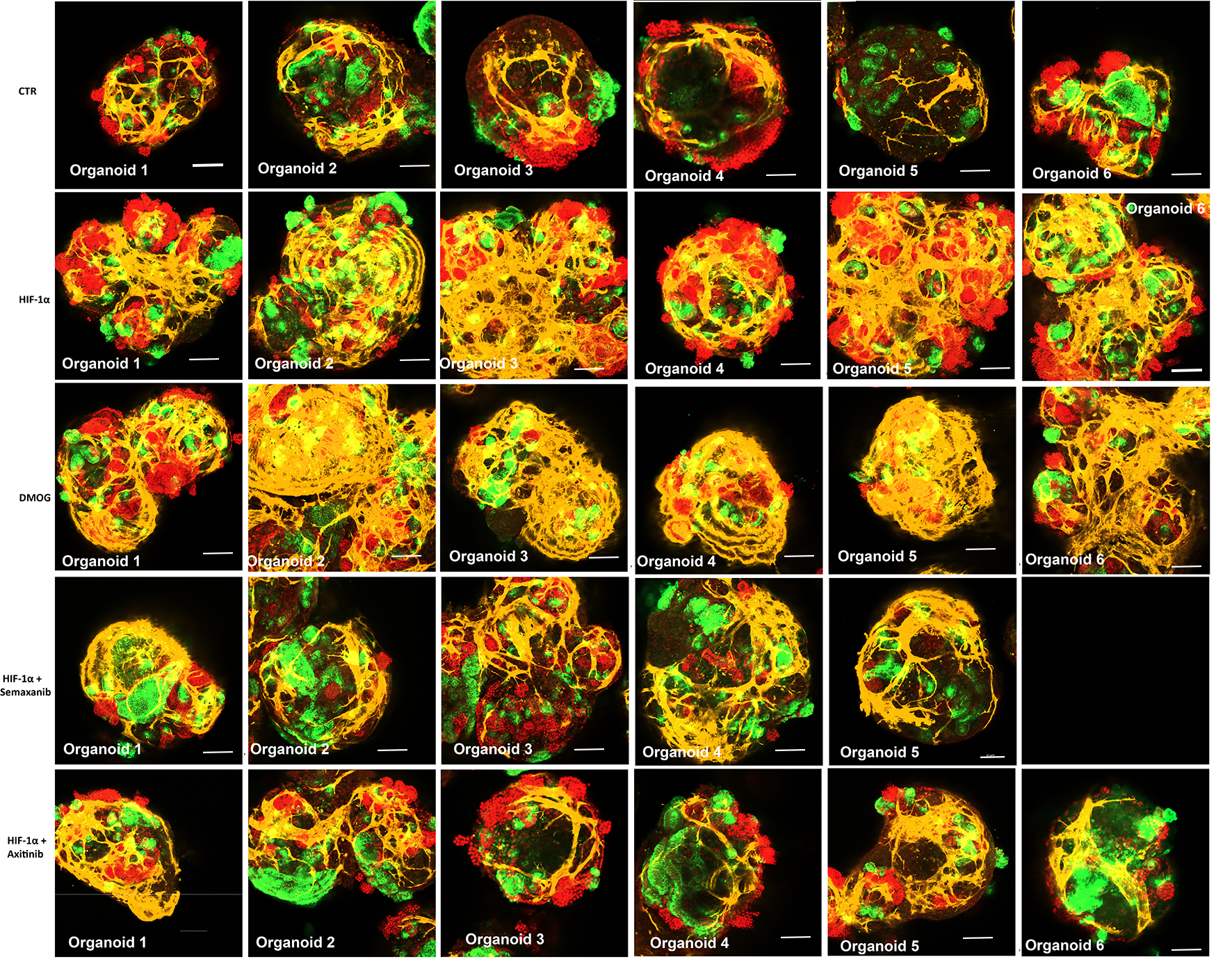

Supplement: Supplementary file 2 — Additional file 2: Fig. S2. The movie for 3D vascularized kidney organoid with HIF-1α overexpression. Z-stack confocal analysis were done for vascularized kidney organoid with podocytes (Wilms' Tumor, yellow), endothelial networks (CD31, red) and proximal tubule (LTL, green). [file 13287_2023_3528_MOESM2_ESM.tif]

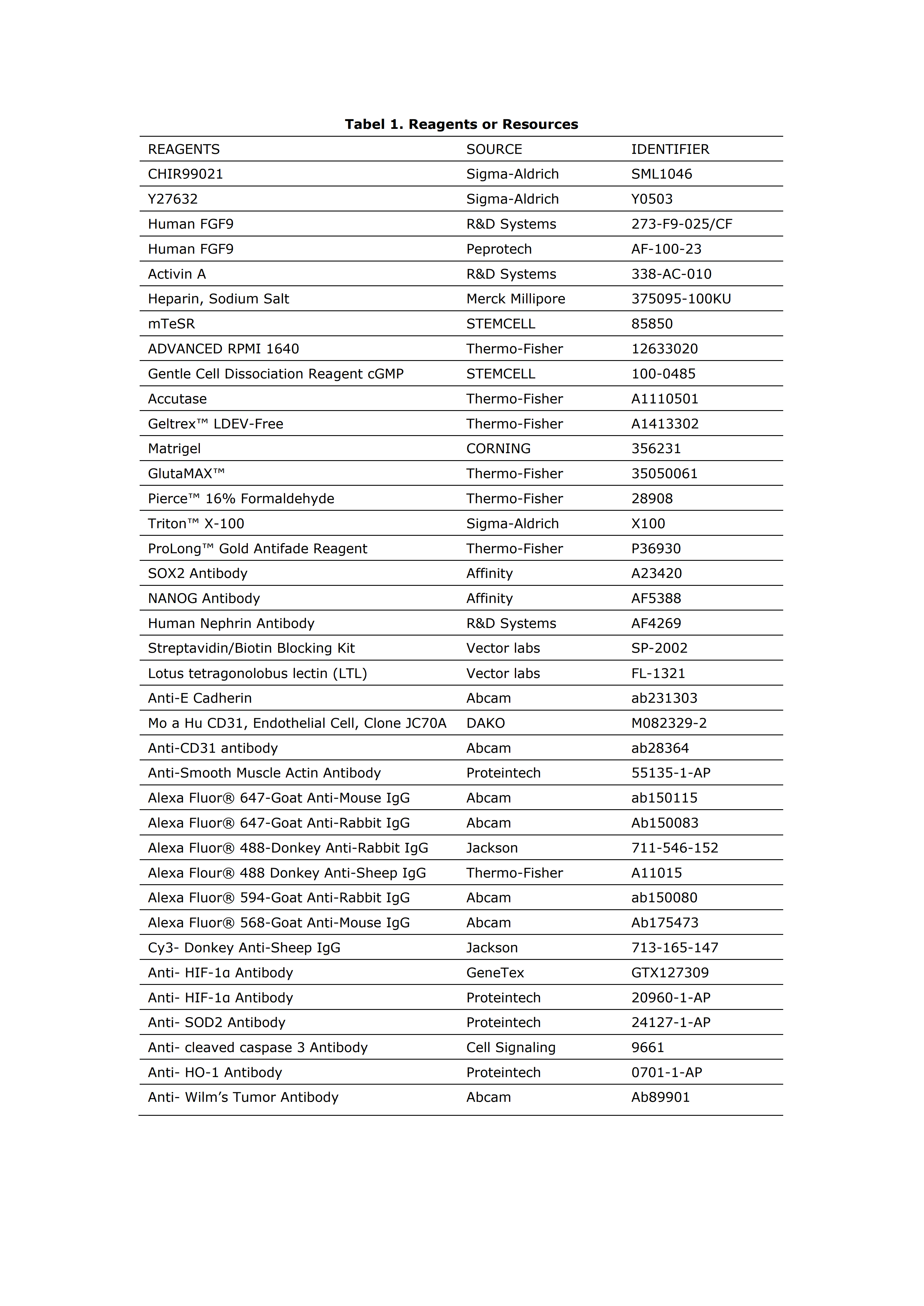

Supplement: Supplementary file 3 — Additional file 3: Table S1. The details of the companies and catalog number of cell culture materials and antibodies. [file 13287_2023_3528_MOESM3_ESM.png]
